# Open Straightening and Direct Puncture of the Superficial Temporal Artery for Tumor Embolization via Transosseous Feeders

**DOI:** 10.7759/cureus.108739

**Published:** 2026-05-12

**Authors:** Makoto Yamada, Naokado Ikeda, Masahiro Kawanishi, Ryusuke Ogawa, Kazuyuki Tane

**Affiliations:** 1 Neurosurgery, Takeda General Hospital, Kyoto, JPN; 2 Neurosurgery, Tane General Hospital, Osaka, JPN

**Keywords:** direct puncture, glue, superficial temporal artery, transosseous feeder, tumor embolization

## Abstract

Direct access to the superficial temporal artery (STA) has traditionally been performed using an open approach; however, ultrasound-guided percutaneous puncture has recently become widely used in several settings. In meningiomas supplied by transosseous STA feeders, stable navigation of a microcatheter beyond the bony penetration site into the dural tumor feeder is essential. Severe tortuosity and sharp angulation near the cranial entry point may render percutaneous approaches unreliable.

To describe an open technique combining STA straightening and a wedged 18-gauge peripheral intravenous catheter (PIVC) used as a sheath substitute to facilitate secure microcatheter passage across the transosseous segment.

The STA was exposed through a skin incision and straightened by removing surrounding connective tissue. A PIVC was inserted via subcutaneous tunneling from a puncture site lateral to the incision and wedged at the cranial entry point. A Marathon microcatheter (Medtronic, Minneapolis, MN, USA) was advanced through the PIVC into the dural feeder, and tumor embolization was performed using N-butyl cyanoacrylate (NBCA).

The microcatheter successfully traversed the transosseous segment and reached the dural tumor feeder. Effective intratumoral embolization was achieved with 12.5% NBCA (1.09 mL), without reflux into cutaneous branches or skin ischemia.

In meningioma embolization requiring reliable use of transosseous feeders, open STA straightening with direct puncture using a wedged PIVC provides secure passage across the cranial entry point and stable catheter control when percutaneous approaches are insufficient, and may be valuable in carefully selected cases.

## Introduction

Direct superficial temporal artery (STA) access has traditionally been performed using an open approach, whereas ultrasound-guided percutaneous puncture is now widely used in several settings [1-3]. In meningiomas supplied by transosseous STA feeders, reliable microcatheter passage beyond the cranial penetration site into the dural feeder is essential but may be hindered by severe tortuosity and sharp angulation. Here we describe an open technique that combines STA straightening and a wedged 18-gauge peripheral intravenous catheter (18G-PIVC) used as a sheath substitute to facilitate stable intracranial catheterization and embolization.

## Technical report

A 63-year-old woman presented with a hypervascular meningioma located at the parietal convexity. Preoperative tumor embolization was required prior to surgical resection. Cerebral angiography demonstrated that the dominant arterial supply was a transosseous feeder arising from the anterior branch of the STA, penetrating the skull and supplying the outer dural surface of the tumor.

Approaches via the external carotid artery trunk or the middle meningeal artery were limited by small caliber vessels, rendering the STA route essentially the only feasible access.

**Figure 1:**
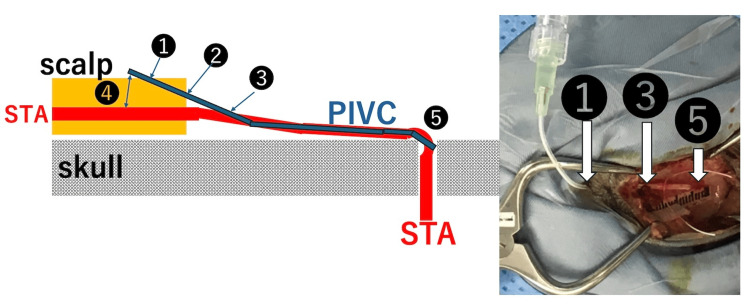
Schema and surgical field photograph of direct puncture Schematic illustration showing the puncture site on the skin (1), subcutaneous tunneling (2), STA cannulation site (3), the angle between the PIVC and the skull (4), and the cranial entry point (5). STA: superficial temporal artery, PIVC: peripheral intravenous catheter

In this technique, a PIVC is used as a sheath substitute to optimize the puncture angle and device stability, thereby facilitating microcatheter navigation across the transosseous segment (Figure 1).

PIVC placement consists of three steps: (1) skin puncture adjacent to the incision edge, (2) subcutaneous tunneling, and (3) direct visual cannulation of the exposed STA after cut-down. This configuration avoids an excessively steep trajectory against the skull and provides fixation at the skin edge, reducing the risk of dislodgement during device manipulation and limiting the need for manual stabilization under fluoroscopy.

Whenever feasible, a long straight working segment between the STA cannulation site and the cranial entry point should be secured. Because the transosseous segment is relatively immobile, distal advancement of the PIVC should be gentle and stopped immediately if resistance is encountered.

Procedural details

Skin Incision, STA Exposure, and Straightening

**Figure 2:**
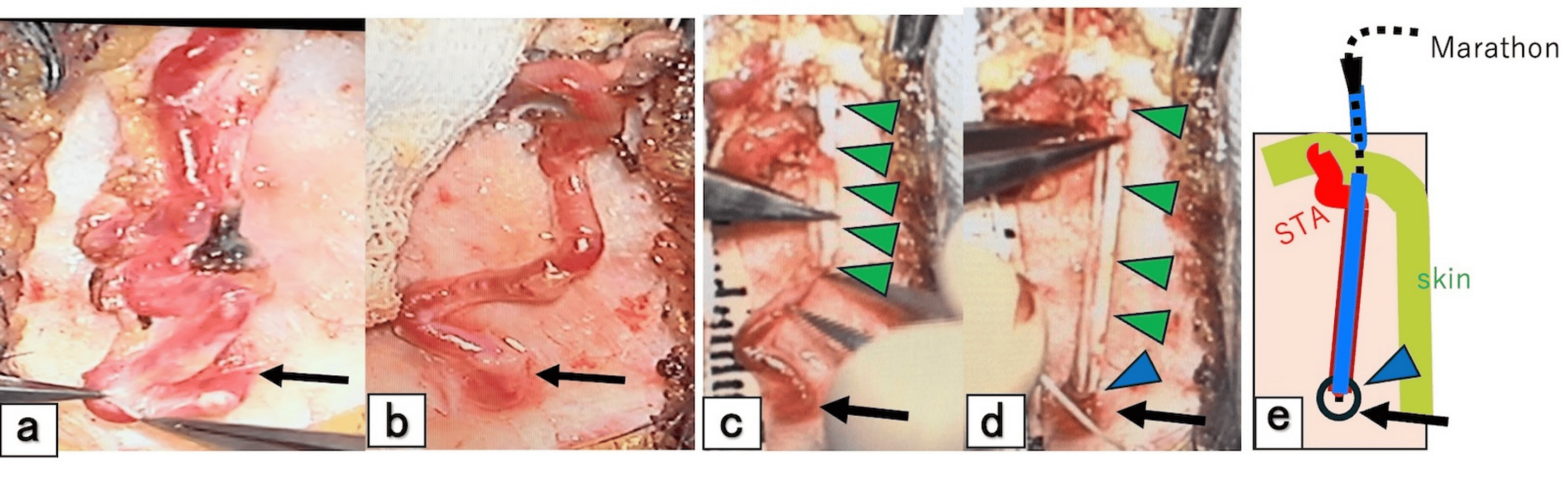
Intraoperative microscopic view of the superficial temporal artery (STA) The black arrow indicates the transosseous entry point through the skull. a. The STA after division of a small branch; surrounding connective tissue remains, and the artery is still tortuous. b. The STA after removal of the surrounding connective tissue, with complete release of tortuosity and straightening of the vessel. c. An 18-gauge peripheral intravenous catheter being inserted into the straightened STA (green triangle). d. The tip of the 18-gauge peripheral intravenous catheter is wedged at the transosseous entry point of the STA (blue triangle), providing stable support for intracranial catheterization. e. Schematic illustration of the microsurgical procedure. The blue structure represents the 18-gauge peripheral intravenous catheter, which is inserted into the STA (red) through a puncture site slightly lateral to the surgical wound with subcutaneous tunneling,and wedged at the cranial entry point (black circle). The Marathon microcatheter is shown advancing through the 18-gauge peripheral intravenous catheter.

A 5-6 cm linear skin incision was made along the STA course, and the artery was exposed. After division of small branches, surrounding connective tissue was carefully dissected to mobilize the vessel and release tortuosity. Particular attention was paid to the segment immediately proximal to the cranial penetration site, where near-skeletonization was performed to align the puncture site and bony canal in a straight trajectory (Figure 2A, 2B).

Direct Puncture and PIVC Placement With Subcutaneous Tunneling

The PIVC was introduced from a puncture site slightly lateral to the wound edge and advanced via subcutaneous tunneling to the exposed STA (Figure 1, Figure 2E). The straightened STA was punctured, and the PIVC was advanced and gently wedged at the cranial entry point (outer table side) to provide stable support and prevent displacement (Figure 2C-2E). 

Microcatheter Navigation to the Dural Feeder

**Figure 3:**
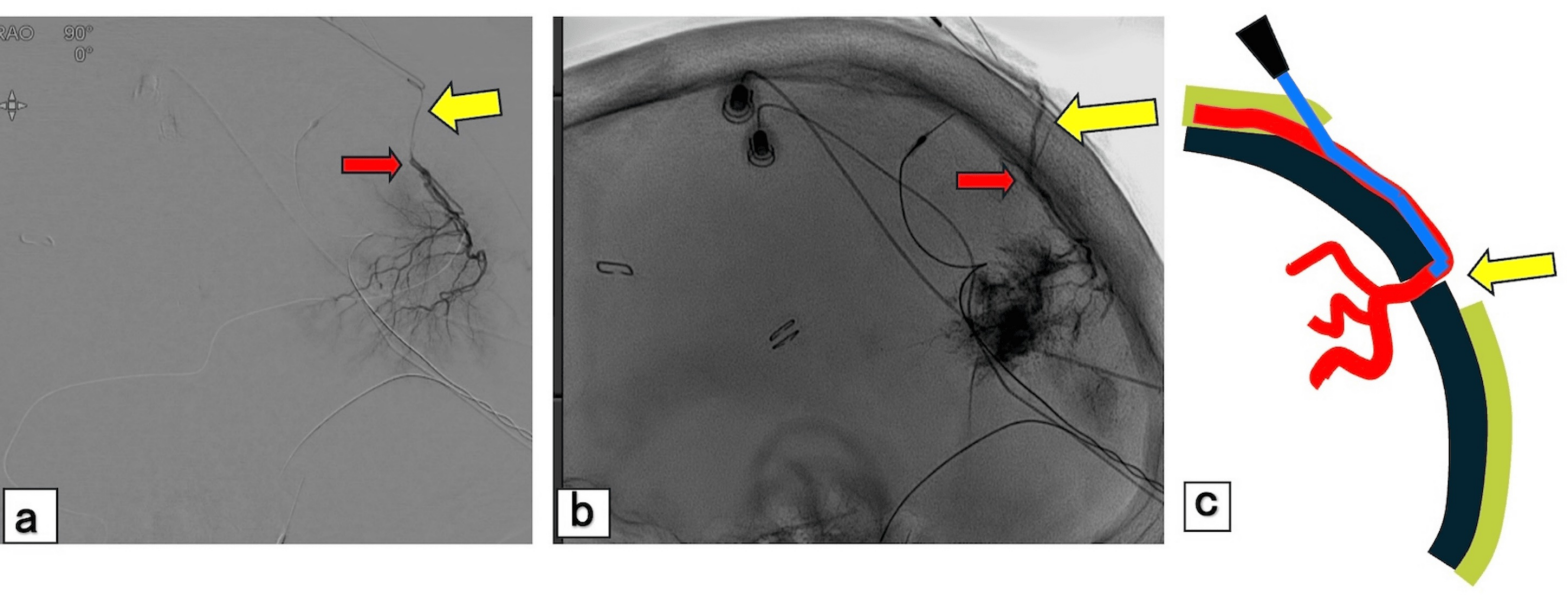
Lateral selective angiography of the parietal region The yellow arrow indicates the tip of the 18-gauge peripheral intravenous catheter wedged at the cranial entry point, and the red arrow indicates the tip of the microcatheter. a. Selective tumor opacification obtained from the microcatheter positioned intracranially. b. Tumor embolization via the transosseous STA feeder. c. Schematic illustration showing the 18-gauge peripheral intravenous catheter (blue) inserted into the STA (red) and wedged at the cranial entry point (yellow arrow). The black line represents the skull, and the green area represents the skin. STA: superficial temporal artery

A guidewire was advanced through the PIVC across the transosseous segment, followed by a flow-directed microcatheter (Marathon; Medtronic, Minneapolis, MN, USA). The microcatheter traversed the cranial penetration site smoothly and was stably positioned within the dural tumor feeder (Figure 3A, 3C).

Embolization

With the microcatheter positioned in the dural tumor branch, 1.09 mL of 12.5% N-butyl cyanoacrylate (NBCA) was injected, achieving effective intratumoral embolization (Figure 3B). No reflux into cutaneous branches or skin circulation disturbances were observed.

## Discussion

Percutaneous STA access has been applied in various fields, including carotid artery stenting [4]; however, traversing the cranial bone is not the primary objective in such procedures. In contrast, parasagittal, interhemispheric, and high-convexity meningiomas are frequently supplied by multiple transosseous feeders arising from bilateral STAs or occipital arteries. These branches are characterized by subcutaneous tortuosity, sharp angulation near the cranial entry point, and step-offs within the bone canal, all of which hinder microcatheter manipulation.

The STA and occipital artery have numerous cutaneous branches; therefore, incomplete embolization of the transosseous feeder may divert flow to the scalp and increase the risk of cutaneous ischemia or necrosis [5]. To minimize this risk, several adjunctive strategies have been reported, including manual compression of cutaneous branches, supportive catheter systems, and percutaneous direct puncture. However, when severe tortuosity exists immediately proximal to the cranial entry point, percutaneous puncture may be unreliable or impossible; in such cases, an open approach prioritizing straightening and stability is rational.

The key feature of the present technique lies in open straightening of the STA, wedging the PIVC at the cranial entry point, and stable advancement of a microcatheter to the dural feeder. This invasive and complex “hybrid technique” is considered for cases involving large convexity lesions with the STA as the main feeder (radial or femoral access is difficult); there are certain tips to perform it safely and reliably in practice.

## Conclusions

In meningiomas predominantly supplied by transosseous STA feeders, reliable microcatheter navigation beyond the cranial penetration site is essential for successful embolization and avoidance of complications. Open exposure and straightening of the STA with direct puncture using a wedged PIVC provide secure access and stable catheter control when percutaneous approaches are insufficient. This technique tip is a valuable option in carefully selected cases.
